# Spontaneous Rabies

**Published:** 1876-05

**Authors:** 


Spontaneous Rabies.—Mr. Fleming, in a recent number of the *Veterinary Journal* (London) reports a case which bears somewhat upon the spontaneous origin of rabies. A retriever dog, which had always been carefully tended, and was never out alone, was on but one occasion attacked by another dog, healthy at the time and since, and in the course of a month afterwards was seized with all the symptoms of hydrophobia, and was killed. While suffering from the disease the retriever bit three other dogs, one of which was seized with "dumb madness," and was eventually killed. The original biter is still alive and well, but under observation. No one doubts Mr. Fleming's ability to diagnosticate hydrophobia, but it is barely possible that the retriever dog may have been bitten by an actually rabid animal, the fact being unknown even to his watchful master.—*Med. Record.*

